# Recent advances in allergic rhinitis

**DOI:** 10.12688/f1000research.15367.1

**Published:** 2018-08-23

**Authors:** Flavia C. L. Hoyte, Harold S. Nelson

**Affiliations:** 1 Department of Medicine, Division of Allergy/Immunology, National Jewish Health, Denver, CO, 80206, USA

**Keywords:** Allergic Rhinitis, Local nasal allergy, Immunotherapy, SCIT, SLIT

## Abstract

Allergic rhinitis affects 20 to 30% of adults in both the United States and Europe and perhaps a somewhat higher percentage of children. In addition to nasal and ocular symptoms directly related to the allergic process, interference of these symptoms with sleep leads to daytime sleepiness and impaired quality of life. Patients miss work because of symptoms but an even greater problem is interference with work productivity, or presenteeism, which has been reported to be the biggest contributor to the total economic cost of allergic rhinitis. There has been increasing awareness that many patients with either seasonal or perennial symptoms but negative skin and *in vitro *tests for allergen sensitivity have local nasal allergy, diagnosable by the presence of allergen-specific IgE in their nasal secretions or a positive nasal allergen challenge or both. The pharmaceutical management of allergic rhinitis rests on symptomatic treatment with antihistamines that perhaps are more effectively administered intranasally than orally and intranasal corticosteroids. Allergen immunotherapy is very effective, even for local allergic rhinitis, and the shortcomings of subcutaneous immunotherapy of inconvenience and safety are reduced by the introduction of sublingual immunotherapy (SLIT). Use of the latter is currently somewhat limited by the lack of appropriate dosing information for SLIT liquids and the limited number of allergens for which SLIT tablets are available.

## Introduction

Allergic rhinitis (AR) is a common condition. Estimates of its prevalence vary widely but good epidemiologic studies suggest that 20 to 30% of adults and up to 40% of children are affected ^1^. Symptoms can have significant negative impact on the patients’ quality of life, often interfere with sleep, and contribute to poor performance at work and school. In approaching the patient with rhinitis symptoms, clinicians must distinguish AR from non-AR (NAR) and nasal symptoms due to mechanical factors but not miss the presence of local nasal allergy. Treatment for more severe disease should employ anti-inflammatory as well as symptomatic medication, and allergy immunotherapy (AIT) should be strongly considered for not only its effectiveness but also its disease-modifying effects. The main challenges in AR relate to its treatment. Symptomatic and topical anti-inflammatory medication is often not fully effective, and AIT can be inconvenient and expensive, and there is much room for improvement in both forms of treatment.

## The burden of allergic rhinitis

In 2004, a mail survey in the US elicited responses from two thirds contacted ^2^. Of 19,678 adult responders, 44.3% reported nasal symptoms on at least seven days per year, 30.2% attributed these symptoms to allergies, and 20.7% reported a physician diagnosis of nasal allergies. In 2001, a two-step survey was conducted in Belgium, France, Germany, Italy, Spain, and the UK ^3^. A questionnaire was administered by telephone to 9646 adults to determine the presence of a diagnosis of or symptoms suggestive of AR. Self-awareness of AR was reported by 19%, and 13% reported a physician diagnosis of AR. All of those with positive responses were invited to a clinical center for definitive diagnosis and 725 were examined. On clinical examination, 411 out of 725 were diagnosed as having AR; 45% of these 411 had not received a previous physician diagnosis. This led to estimates of clinically confirmable AR varying from 17% in Italy to 29% in Belgium and overall 23% for the European population studied. The incidence and prevalence of AR in the children were studied in an Isle of Wight birth cohort (n = 1456) recruited in 1989 ^4^. Prevalence of AR increased from 3.4% at 4 years of age to 27.3% at 18 years and was more common in boys than girls at that age.

In a telephone survey of 2765 adults and children (at least five years old) with a diagnosis of nasal or ocular allergies or both, 78% reported seasonal symptoms, and the peak was during the tree season (March to May) and a lesser peak occurred in the fall weed season (September) ^5^. At the peak of their or their child’s allergy season, 39% rated nasal congestion and 34% red itchy eyes as “extremely bothersome” whereas 29% said daily life was “impaired a lot” ^5^.

The symptoms of AR interfere with the ability to sleep, leading to daytime sleepiness and impaired quality of life ^6^. In a survey of 100 patients who had moderate/severe AR, sleep disturbances were reported by 66% of adults and 43% of children ^7^. Patients with moderate/severe AR, compared with those with mild disease, had significantly more anxiety, depression, fatigue, trouble with social interactions, and perceived signs of cognitive dysfunction ^7^. The disturbances of sleep extend to parents of children with AR, 75% of whom reported poor-quality sleep ^8^.

A Medline search retrieved original studies from 2005 to 2015 on the impact of AR on work productivity ^9^. Pooled analysis of studies in which the validated Work Productivity and Activity Impairment (WPAI) questionnaire had been used to collect data found an estimated 3.6% of missed work time (absenteeism) and 35.9% of work performance impairment (presenteeism) due to AR. The cost of absenteeism and presenteeism was estimated to be 3.2- to 13.5-fold higher than direct medical costs and to represent 76 to 93% of the total costs of AR ^9^. School performance is also affected by AR. In a case control study in the UK, students who were 15 to 17 years of age and currently symptomatic with AR were significantly more apt to have lower examination scores in the summer compared with the winter ^10^. The cost of AR was assessed in a representative sample of the Swedish population (18 to 65 years of age) in a report published in 2016 ^11^. The mean annual direct and indirect costs because of AR were 210 Euros and 750.8 Euros, respectively. Of the total cost, 8.1% was due to absenteeism and 70.0% was due to presenteeism. The remainder was equally divided between pharmaceutical and health-care costs ^11^. The cost for the European Union countries for absenteeism and presenteeism caused by AR in untreated or inadequately treated individuals has been estimated at 55 to 151 million Euros per year ^12^.

## The impact of climate change on allergic rhinitis

The potential effect of climate change on the severity and extent of AR has been examined, most intensively in the case of ragweed pollen ^13– 15^. Increasing temperature and carbon dioxide exposure have been shown to increase the production of pollen from individual plants ^13^. At the same time, the increase in the number of frost-free days and the later occurrence of the first frost have been shown to correlate with longer ragweed pollen seasons and are predicted to allow ragweed to propagate further north ^14^. This is of special concern in Europe, where ragweed is established in the Rhone Valley/Burgundy in France, northern Italy, Hungary, and surrounding countries ^15^. Thus, with favorable climatic conditions, it is poised to extend into Poland, Germany, and northern France ^15^.

## Rhinitis subtypes

The first step in rhinitis management is determining the type(s) of rhinitis that an individual has and this can be complicated given the various phenotypes and endotypes of rhinitis. A phenotype is defined by clinical presentation, whereas an endotype is defined by underlying pathophysiologic mechanism. AR is generally differentiated from NAR by the presence of positive allergy skin testing or serum-specific IgE testing. As discussed below, there has been an increasing interest recently in local AR (LAR), which is generally diagnosed in individuals with negative serum and skin allergy tests but histories suggestive of AR, through a positive nasal allergen challenge or the identification of specific IgE (sIgE) in nasal secretions or both ^16^.

NAR has many subtypes, including infectious, drug-induced, gustatory, hormone-induced, atrophic, senile, and idiopathic rhinitis (IR) ^17^. Nasal symptoms can also occur as a result of structural or mechanical issues, such as choanal atresia, adenoidal hypertrophy, septal deviation, nasal tumors, or cerebrospinal fluid leaks, as well as systemic conditions, such as cystic fibrosis, primary ciliary dyskinesia, eosinophilic granulomatosis and polyangiitis, sarcoidosis, and amyloidosis. Occupational rhinitis, which can be either allergic or non-allergic, has many causative agents and can present either shortly after starting an occupation with a new antigenic exposure or following a latency period, when an individual is developing sensitization to the new antigen. Oftentimes, patients have more than one type of rhinitis, leading to a mixed phenotype or endotype or both ^16, 18^.

## Natural history of allergic rhinitis

The natural history of AR seems to be different from that of NAR ^19^. Young children with NAR are more likely to go into remission than their AR counterparts. In a birth cohort of over 2000 children, 73% of NAR subjects and only 12% of AR subjects went into remission between ages 4 and 8. The proportion of children with NAR decreased slightly from age 4 (8%) to age 8 (6%), whereas AR rates increased between these ages from 5% to 14%. Sensitization often precedes AR as over half of those who were sensitized but asymptomatic at age 4 developed AR by age 8 ^19^. In a questionnaire-based study of adults who were 20 to 59 years old, about 23% of cases demonstrated remission of their AR symptoms within an eight-year period from 1992 to 2000, and the highest rate of remission was in the oldest age group (50 to 59 years) and the lowest rate of remission was in those with concomitant asthma. The highest incidence of new-onset AR was seen in the youngest age group (20 to 29 years) ^20^.

ARIA (Allergic Rhinitis and its Impact on Asthma), the World Health Organization initiative on AR, published guidelines in 2001 that shifted the paradigm from classifying AR as either seasonal AR (SAR) or perennial AR (PAR) to a classification based on frequency (persistent or intermittent, as defined below) and severity (mild or moderate/severe, based on whether or not there is impaired sleep, impairment of daily life, or troublesome symptoms) ^21^. They defined intermittent AR as affecting patients less than four days a week or less than four consecutive weeks, whereas symptoms in persistent AR lasted more than four days a week or more than four consecutive weeks ^21^. Since their initial publication, these guidelines have been updated and refined multiple times ^22– 24^.

Based on a study from Western Europe in which telephone interviews were performed on 9646 individuals, 726 of whom came to the clinical center for an evaluation, the investigators found a poor correlation between the seasonal/perennial terms and the intermittent/persistent classifications, respectively, and about half of the persistent subjects had SAR and half of the intermittent subjects had PAR ^3^. Another study, by Ciprandi *et al*., demonstrated that 80% of patients with AR have a mixed form of SAR and PAR, suggesting to the authors that the terms PAR and SAR are poorly reflective of real life and that intermittent and persistent may be more applicable ^25^.

## Local allergic rhinitis

In the last decade and a half, there has been increasing interest (particularly by a group led by Miguel Blanca in Malaga, Spain) in LAR, a term applied to patients whose allergy skin and blood testing is negative but who have a history suggestive of allergic sensitization and local evidence of atopy diagnosed by sIgE in nasal secretions or by positive nasal allergen challenge or both.

In a study of 50 patients with perennial NAR (PNAR), 30 with PAR, and 30 healthy controls, Rondón *et al*. found a similar nasal leukocyte-lymphocyte phenotype in the nasal lavage of PAR and PNAR patients, both of which differed from normal controls, who tended to have fewer eosinophils, total lymphocytes, and CD3 ^+^CD4 ^+^ lymphocytes ^26^. A positive nasal challenge with *Dermatophagoides pteronyssinus* (D. pt.) was present in 54% of the patients with PNAR, and sIgE was identified in 22% of these individuals ^26^. In another study, in which 40 patients with LAR due to D. pt. underwent nasal challenge with D. pt., 60% had isolated and 40% had dual positive responses ^27^. Tryptase was present in the nasal secretions of 45%, elevated eosinophil chemotactic factor in 65%, and D. pt.–specific IgE in 25%. LAR to pollens has also been demonstrated by this group in a similar study comparing individuals with seasonal IR with those with pollen-induced AR. This study found similar nasal leukocyte-lymphocyte profiles in both sets of rhinitis patients, which were different from that of controls. Of the subjects with IR, 62.5% had a positive nasal allergen provocation test (NAPT) and 35% demonstrated nasal sIgE ^28^. Rondón, *et al*. also have investigated multiple NAPT (NAPT-M), in which four different allergen extracts are administered at 15-minute intervals, and have demonstrated 100% concordance with single-aeroallergen NAPTs, suggesting that this may be an efficient and cost-effective way to detect polysensitization in patients with LAR ^29^.

A study to determine the prevalence of LAR in a Spanish population evaluated 452 unselected adult patients with rhinitis by means of skin-prick tests, sIgE, and NAPT; they diagnosed LAR in 25.7%, AR in 63.1%, and NAR in 11.2% ^30^. A study evaluating LAR to house dust mites (HDMs), specifically evaluating a pediatric population with NAR (mean age of 11 years and median disease duration of 6.3 years), used nasal tryptase, symptoms, physiologic measures (peak nasal inspiratory flow and acoustic rhinometry), and local production of HDM-sIgE and demonstrated that LAR to HDM is rare in children. Only two (3.7%) of the 54 children showed significant change in symptoms and physiologic measures following HDM-NAPT ^31^. In contrast, a study by Bozek *et al*. looking at an elderly population (mean age of 65.81, n = 219) with undiagnosed persistent rhinitis demonstrated a rate of LAR of 21%, and D. pt. was the main sensitizing aeroallergen in those with LAR (63%) ^32^. In this study, symptom changes during NAPT were accompanied by an increase in nasal sIgE. The large, questionnaire-based studies conducted to determine the prevalence of AR in the general population could not assess the prevalence of LAR since nasal allergen challenges were not part of the protocol ^2, 3^.

A five-year follow-up study in 194 patients with LAR demonstrated worsening in 26.3% of the patients, and the development of atopy at five years was detected by skin-prick test or sIgE (or both) in 6.81% of LAR and 4.5% of normal control patients ^33^. At 10 years, the rate of development of systemic atopy was similar in the LAR patients and controls (9.7% versus 7.8%, *P* = 0.623). This indicates that LAR is usually a persistent condition and not a precursor to AR. The patients with LAR in this study demonstrated a significant worsening of their rhinitis over time and this was clinically relevant and associated with development of asthma ^34^. Treatment of LAR with immunotherapy is discussed in the section on immunotherapy, below.

## Management of allergic rhinitis

Management of AR continues to revolve around allergen avoidance, medications that provide symptomatic relief, anti-inflammatory therapies, and AIT. Recent advances in therapy include intranasal antihistamines and novel methods of delivery for intranasal steroids, which continue to be the mainstay of therapy for AR and now can be found over-the-counter in the US for some formulations.

Intranasal antihistamines, introduced in the US in 2000 ^35, 36^, have broadened the landscape of intranasal medications for the treatment of AR. They have been shown to improve the total nasal symptom score (TNSS) as well as each individual nasal symptom score (INSS) for sneezing, rhinorrhea, nasal congestion, and nasal itching with faster onset and similar efficacy when compared with intranasal steroids ^37^. Intranasal antihistamines also have a faster onset than oral antihistamines and can improve all INSSs to a similar degree, except for nasal congestion, which is better controlled by nasal compared with oral antihistamines ^38^.

Novel delivery methods of intranasal steroids include a nasal preparation of ciclesonide, a steroid pro-drug that is converted to its active form only upon tissue delivery, introduced in aqueous form in the US in 2006 ^39^; a mist formulation of fluticasone furoate, approved by the US Food and Drug Administration (FDA) in 2007 ^40^; and aerosol devices using hydrofluoroalkane (HFA) propellant to deliver beclomethasone ^41^ and ciclesonide ^42^, both approved by the FDA in 2012. The newest delivery mechanism for intranasal therapy is an exhalation-activated device, which currently (2017) is approved only for the delivery of fluticasone propionate to treat nasal polyps ^43^ but which eventually might be approved for the treatment of AR.

In 2012, a combination spray containing both fluticasone propionate and azelastine hydrochloride was approved by the FDA ^44^. Aggregate data from 3398 subjects in three multicenter, randomized, double-blind, placebo- and active-controlled, parallel-group trials demonstrated a faster onset and superior efficacy as compared with each of the individual components. The onset of action was 30 minutes, and clinical improvement was observed during the first day of assessment and there was sustained benefit for the entire two-week study period ^45^. An updated consensus treatment algorithm proposes an approach to treatment that is in keeping with the ARIA guidelines and highlights a more prominent role for nasal antihistamines ^46^.

Although there has been a recent increase in interest among the general population regarding complementary and alternative medicine (CAM) approaches, including acupuncture, traditional Chinese medicine, and homeopathy, there is a paucity of randomized controlled studies evaluating these approaches. Acupuncture has the greatest amount of data and shows promise in smaller randomized studies ^47– 50^. Several review articles have been published on the topic of CAM and its role in treating allergic disease, including AR ^51– 53^. The consensus among these articles is that although CAM is considered to be low-risk and to have potential benefit, additional studies are needed to fully evaluate the efficacy and potential long-term benefit of these therapies ^51– 53^.

## Efficacy of subcutaneous and sublingual immunotherapy in allergic rhinitis

A number of systematic reviews (SRs) of subcutaneous immunotherapy (SCIT) or sublingual immunotherapy (SLIT) (or both) for AR have been conducted. A committee of the European Academy of Allergy and Clinical Immunology critically assessed these SRs for evidence of the effectiveness, safety, and cost-effectiveness of AIT for allergic rhinoconjunctivitis (ARC) ^54^. They identified 17 SRs published through the end of October 2015. These SRs suggested that, in carefully selected patients, SCIT and SLIT resulted in significant reductions in symptom scores and medication requirements for ARC with reassuring safety data. The data could not support conclusions on the relative clinical effectiveness or the cost-effectiveness of the two approaches to AIT.

The major recent development in AIT in the US for ARC has been the introduction of SLIT tablets containing Timothy grass, a five-grass mixture, short ragweed, or HDM (D. pt. and *Dermatophagoides farinae*) extracts. The doses of the five tablets chosen for commercial development, expressed as the content of major allergen, are listed in Table 1. In most, a dose-ranging study has been performed that identified an effective dose as well as one or more less effective doses ^55– 59^. In the case of ragweed and HDMs, there were also studies that suggested that doses higher than those eventually approved carried safety concerns ^60, 61^.

**Table 1.** Major allergen content of sublingual immunotherapy tablets selected for commercial development.

Author	Allergen extract	Major allergen content	Clinical effect versus placebo
Durham *et al*. ^55^ (2006)	Timothy	75,000 SQ = 17 μg Phl p 5	Symptoms 21% ^a^ Medications 29% ^a^
Didier *et al*. ^56^ (2007)	Five grasses (orchard, meadow, perennial rye, sweet vernal, and Timothy)	300 IR = 25 μg group 5 allergen	RTSS 300 IR 37% 500 IR 35%
Nolte *et al*. ^57^ (2013) Creticos *et al*. ^58^ (2013)	Short ragweed	12 Amb a 1-U = 12 μg Amb a 1	TCS Peak ragweed season: 27% and 24%
Demoly *et al*. ^59^ Nolte *et al*. ^62^	House dust mites	12 DU = 7.5 μg each of Der p 1, Der f 1, Der p 2, and Der f 2	6 DU – TCS 17.3% 12 DU – TCS 17.7% 12 DU – TCS 17%
Bergmann *et al*. ^63^ (2014) Okamoto *et al*. ^64^ (2016)	House dust mites	300 IR = 16 μg Der p 1+ 68 μg Der f 1	AAdSS 300 IR – 17.9% 500 IR – 20.2% AASS 300 IR – 18.2% 500 IR – 13.1%

^a^Subjects who completed at least eight weeks of treatment before the grass pollen season. AAdSS, average adjusted symptom score (symptom score adjusted for medication use); AASS, average adjusted symptom score (adjusted for medication use); DU, developmental units; IR, index of reactivity; RTSS, rhinoconjunctivitis total symptom score; SQ, standardized quality; TCS, total combined score.

The safety data in these phase III studies of SLIT tablets have been reassuring, and no fatal or life-threatening adverse reactions have been reported ^55– 59, 64^. The use of epinephrine to treat SLIT tablet-related adverse events (TRAEs) was reported for 29 studies conducted for the registration of the ALK Timothy, short ragweed, and HDM SLIT tablets ^65^. Epinephrine was administered to treat 16 TRAEs: six systemic and 10 local application site reactions. Of the six systemic reactions, none were considered serious, five occurred with the first administration under physician observation, and one occurred on the sixth day of treatment. The occurrence of adverse reactions to short ragweed SLIT tablet was tabulated for the first 28 days of four safety or efficacy studies (or both) conducted for registration of the product ^66^. Local application site reactions were common but usually mild-moderate, of brief duration, and occurring mostly in the first week or two of treatment.

Five-year studies were conducted with both the Timothy ^67^ and the five-grass ^68^ SLIT tablets, in which treatment administered either continuously ^67^ or pre- and co-seasonally ^68^ was continued for three years, and the subjects were followed for two years after cessation of treatment to determine whether there was persisting benefit. With the Timothy tablet, there was 36% improvement compared to placebo in the symptom/medication score during the third year of treatment and persisting improvement of 34% and 27% the two follow-up years ^67^. With the five-grass SLIT tablet, the improvement in the symptom/medication score during the third year of treatment was 39%, and that the two follow-up years was 30% and 28% ^68^. Both results suggest that there has been modification of the underlying immunologic process by the immunotherapy. On the other hand, a study with the same dose of Timothy SLIT tablet, administered daily but for only two years, produced a clinical response that was lost one year after treatment discontinuation, suggesting that treatment of at least three years was required to produce persisting improvement ^69^.

Further evidence of disease modification by AIT comes from the results of a phase IV study with the Timothy grass SLIT tablet that examined the effect of three years of treatment of children ages 5 to 12 years who had documented grass-induced AR but no evidence of pre-existing asthma on careful screening ^70^. In the 812 children, three years of treatment produced persisting improvement in clinical AR during two years of follow-up. More importantly, the development of both summertime and wintertime asthma was significantly reduced in the group that had received the Timothy SLIT tablets when compared with those who had received placebo treatment ( Table 2). This suggests, for the first time, that immunotherapy may protect against the development of asthma caused by factors beyond the allergen used in treatment.

**Table 2.** Reduction in the incidence of asthma in children treated 3 years with Timothy grass SLIT tablets with 2-year follow-up ^70^.

	Year 1	Year 2	Year 3	Year 4	Year 5
Summer visits					
SQ grass versus placebo SLIT tablet	OR = 0.57 *P* = 0.067	OR = 0.40 *P* = 0.0014	OR = 0.54 *P* = 0.048	OR = 0.37 *P* = 0.00064	OR = −0.55 *P* = 0.042
Winter visits					
SQ grass versus placebo SLIT tablet	OR = 1.69 *P* = 0.13	OR = 1.22 *P* = 0.53	OR = 0.54 *P* = 0.059	OR 0.44 *P* = 0.016	OR = 0.37 *P* = 0.0027

OR, odds risk; SLIT, sublingual immunotherapy; SQ, standardized quality.

Use of SLIT with liquid preparations in the US has been limited by the lack of an FDA-approved allergen extract for SLIT, although there is some “off label” use ^71, 72^. In Europe, SLIT with liquid preparations is widely employed. However, studies of extracts from three major European extract manufacturers suggested widely varying doses, some of which are well below those that have proven to be necessary for clinical efficacy in dose-ranging studies of SLIT tablets ^73^. The same marked heterogeneity of dosing was found in a study of five commercial HDM liquid SLIT preparations in Spain ^74^. The ranges in doses recommended by the five companies were 130-fold for Der p 1, 129-fold for Der f 1, and 115-fold for group 2 allergens. An additional problem with the use of SLIT liquid extracts in the US, where multiple-allergen mixes are routinely employed in SCIT, is the lack of evidence for efficacy of mixtures of more than two unrelated extracts ^75^.

In comparison with SLIT, there have been far fewer recent studies with SCIT. Most have reported on the development of products that offer greater convenience and safety over the use of SCIT with the currently available products. For the most part, these products are still under development. The exceptions are the allergoids, extracts modified by treatment with aldehydes, that in some cases have shown markedly reduced allergenicity, allowing very rapid build-up in dosing ^76^.

AIT has also been used in patients with LAR (see section on LAR above). Thirty-six subjects with perennial symptoms and positive NAPTs to D. pt. received SCIT with D. pt. extract. At the end of 24 months, daily combined symptom/medication scores were reduced 42% compared with placebo ( *P* = 0.001) and NAPT after one year showed markedly increased tolerance for D. pt. allergen ^77^. The same group also conducted a two-year trial of SCIT with a Timothy grass allergoid extract in 56 LAR subjects with positive NAPT to Timothy ^78^. In the first grass pollen season, after about six months of SCIT, combined symptom medication scores were reduced 54%. The efficacy of SCIT in pollen-induced LAR was also demonstrated in a two-year study in 28 subjects with birch sensitivity ^79^. The efficacy of SLIT in patients with LAR has not been reported.

## Concluding remarks

There is no question that AR is a very common and often very burdensome disease. It can be classified as seasonal or perennial, which has the attraction of directing attention to the relevant aeroallergen, or as intermittent and persistent and mild or moderate/severe that reflects more the burden of the disease on the patient. It is important not to confuse NAR with AR but also not to miss LAR if indeed it is as common as reports indicate. There is a need for more effective forms of symptomatic therapy ^80^. There also is a need for new approaches to AIT that overcome the factors of safety and inconvenience with SCIT but also the limited number of allergens with established dosing with SLIT. Both of these forms of AIT involve several years of treatment to produce lasting results and in many patients this leads to poor adherence with the treatment program ^81^. Forms of AIT that require far fewer treatments over shorter periods of time are sorely needed.

## Abbreviations

AIT, allergy immunotherapy; AR, allergic rhinitis; ARC, allergic rhinoconjunctivitis; ARIA, Allergic Rhinitis and its Impact on Asthma; CAM, complementary and alternative medicine; D. pt., *Dermatophagoides pteronyssinus*; FDA, US Food and Drug Administration; HDM, house dust mite; INSS, individual nasal symptom score; IR, idiopathic rhinitis; LAR, local allergic rhinitis; NAPT, nasal allergen provocation test; NAR, non-allergic rhinitis; PAR, perennial allergic rhinitis; PNAR, perennial non-allergic rhinitis; SAR, seasonal allergic rhinitis; SCIT, subcutaneous immunotherapy; sIgE, specific IgE; SLIT, sublingual immunotherapy; SR, systematic review; TRAE, treatment-related adverse event
